# Impact of Neighborhood Environment on Pedestrian Route Selection among Elementary Schoolchildren in Korea

**DOI:** 10.3390/ijerph18137049

**Published:** 2021-07-01

**Authors:** Somin Lee, Myeong-Hun Lee

**Affiliations:** 1 Department of Urban and Regional Development, Hanyang University, Seoul 04763, Korea; somin0626@naver.com; 2 Department of Urban Planning and Engineering, Hanyang University, Seoul 04763, Korea

**Keywords:** neighborhood environment, impact factors, elementary school students, schoolchildren behavior, pedestrian route choice

## Abstract

Previous studies on the walking environment of elementary school students have focused on physical factors such as traffic accidents, safety, and the neighborhood environment. However, scholars have yet to consider the behavioral characteristics of elementary school students, particularly in respect to the relationship between environmental factors and behavioral characteristics in pedestrian route selection and safety. Addressing this gap, this study identifies how neighborhood environment factors and behavioral characteristics impact route selection and satisfaction among elementary school students. Accordingly, this study surveyed 251 elementary school students at three elementary schools in Korea and analyzed the spatial forms of the selected sites. In doing so, this study discerns students’ satisfaction with their selection of the shortest or non-shortest route and which environmental factors and behavioral characteristics influenced their selection and satisfaction. Study results have practical implications for policymaking, including valuable insights into the planning of school routes for elementary school students.

## 1. Introduction

In a society in which automobiles are essential, there is a greater need for urban mobility and convenience. However, the growing number of cars has resulted in issues such as traffic congestion and a lack of parking, leading drivers to utilize residential roads in an attempt to bypass traffic or find a place to park. Such practices have resulted in an increased incidence of pedestrian accidents in residential areas. More specifically, places with a concentration of low-rise dwellings tend to lack parking space. This is because in detached housing districts, land use plans tend to underestimate parking demand, resulting in an insufficient supply of parking lots. Even in planned detached housing districts such as residential development districts, unauthorized parking along the road is a serious problem. This is primarily due to the relative difficulty in securing parking lots based on the number of cars owned. The number of households increased as the houses that were previously planned as low-rise single-family houses were reconstructed into villas over time [1]. Consequently, most roads comprise a mix of vehicle and pedestrian movements—a combination that produces a higher risk of road accidents. Both weaker and smaller than adults, children are particularly vulnerable as there is a greater risk of them not being seen by drivers. According to 2018 statistics from the Korea Transport Institute, 77% of children’s pedestrian traffic deaths occurred in residential areas, with 40% of deaths on back roads [2]. Given the nature of the neighborhood environment, with most schools located in residential areas, any attempt to ensure the safety of children’s routes must take the aforementioned risks into account.

In Korea, a number of projects are actively attempting to solve pedestrian traffic problems in residential areas and ensure children’s pedestrian safety, including the Residential Environmental Improvement Project, CPTED (Crime Prevention Through Environmental Design), the Pedestrian Priority Street Project, and the Safe Routes to School Project. However, these efforts do not appear to have had any significant impact on reducing the number of accidents or deaths in residential areas. Indeed, pedestrians accounted for an average of 40% of all traffic accident deaths between 2015 and 2020, with 1.5 times more accidents involving children under the age of 12 than those involving adults (19 and over) [3]. Children’s traffic accidents appear to increase significantly during the afternoon, with most occurring between 3:00 and 6:00 p.m. [2,3]. Additionally, about 91% of accidents appear to occur outside of children’s protection zones [2,3]. In this respect, current children’s protection zones are concentrated around schools, and thus fail to reflect the characteristics of the urban structure of the residential area or to fully consider the behavioral characteristics and preferences of schoolchildren.

Environmental design and urban structure can play a decisive role in the characteristics of pedestrian movement. Facilities with a design tailored to the environment can encourage pedestrian activities without threatening pedestrian safety and convenience [4,5,6,7]. The spatial structure of the neighborhood zone (urban form) is determined by density, the size of blocks, and horizontal patterns [8,9,10,11]. The smaller the block, the shorter the distance of the block, the more opportunities there are to turn corners, and the less monotonous paths pedestrians have to choose [12,13]. Most pedestrians consciously choose paths [12] depending on several factors, generally preferring the shortest ones [14,15]. Therefore, it is necessary to verify these differences in areas with different urban shapes, densities and block sizes, and street patterns.

In general, children’s daily lives revolve around school and home [13,16,17]. Accordingly, in order to create a safe school commute for elementary school students in a neighborhood environment, it is necessary to consider the criteria by which children select routes. However, while numerous studies have examined the physical situation around elementary schools [18,19,20], few scholars have considered the relationship between the physical environment and the behavioral characteristics of route choice. Although some studies have addressed the characteristics of children’s travel [13,21], it is difficult to generalize from this research due to limited study scopes and small sample sizes. Similarly, while several studies have examined route selection according to the composition of the street network, these have tended to focus on pedestrian networks and have overlooked the characteristics of the urban structures around schools [22]. This study addresses these gaps by examining the relationship between neighborhood environment and behavioral characteristics in the route selection of elementary schoolchildren in Korea.

Significantly, both the urban form of a neighborhood and the impact of neighborhood environment vary from one place to another. For instance, the urban form of a neighborhood will vary according to whether it is a dense or low-density space [23]. Similarly, high-density cities with high accessibility can offer more social opportunities [23,24,25], which, together with the characteristics of individual pedestrians, influences route choices in nearby areas [23,26]. As the neighborhood environment around elementary schools is a living and major activity space for residents, the relationship between various environmental factors—such as the urban form of the surrounding environment and behavior characteristics of pedestrians—is important. Therefore, this study considers the interaction between various factors in the neighborhood environment, such as the surrounding walking environment and route selection.

This study examines the relationship between neighborhood environment factors and the behavioral characteristics of elementary schoolchildren in selecting pedestrian routes. More specifically, this study identifies which factors elementary school students consider important when selecting a route, which factors influence the selection of a detour (i.e., longest route) over the shortest route, and how these factors are influenced by the characteristics of the urban structure of the neighborhood. In doing so, this study provides new insights into children’s route selection, including implications for developing and planning school commute routes for elementary school students.

## 2. Literature Review and Conceptual Framework

### 2.1. Urban Structure and Travel Behavior

Dempsey defines urban form as physical and non-physical characteristics, which can generally be categorized into five broad and interlinked elements: land use, layout, density, housing/building type, and transportation infrastructure [27]. A broader concept than land use, the concept of urban scale combines a number of morphological characteristics, including the attributes of the transportation system and urban design [5,27]. These characteristics range from urban design and density, the size and layout of blocks, sidewalk length and street type, and the accessibility of pedestrian facilities and routes, and can directly and indirectly affect pedestrian traffic and vehicular traffic [5,28].

A number of studies examining the relationship between urban form and walking behavior have underscored the relative importance of various urban form factors within a region and their influence on the characteristics of individual travel choices [5,8,9,17,25,29,30]. In Seoul, housing type is the largest factor determining the urban form [11,31], particularly the ratio of apartments (i.e., density). Accordingly, it is possible to evaluate urban organization and density based on housing type [5,11], which is a major factor determining the type and lifestyle of a dwelling place [31]. Moreover, a wide range of elements comprising the shape of a city (e.g., horizontal patterns, the accessibility of facilities, and pedestrian networks) differ according to its density and the characteristics of its urban organization [23]. Similarly, morphological characteristics—such as the pedestrian environment, walking conditions, and surrounding facilities—can affect travel behavior, route choice, and means of transportation in various ways [25,32,33].

### 2.2. Travel Behavior and Route Selection

#### 2.2.1. Important Factors in Route Selection

In addition to the properties and physical street environment of each pedestrian, a diverse range of factors influence travel behavior, including surrounding land use and residential density, block size and street pattern, the accessibility of facilities, and pedestrian networks [4,6,32,34]. Elementary school students, who spend most of their time in the neighborhood environment, may be directly or indirectly influenced by these factors when choosing a route [17,20]. In cities such as Seoul, density and urban form vary depending on the type of housing in high-density residential environments (i.e., high apartment ratio), influencing the characteristics of urban structures such as street patterns, the accessibility of facilities, and pedestrian networks [5,11,17]. The characteristics of these urban structures often reflect individual differences in the elements of the neighborhood environment [14,29,35,36]. Indeed, a number of studies suggest that route choice is influenced by attributes of the neighborhood environment in a variety of ways [23,30,37]. These characteristics also impact which factors influence the travel behavior of schoolchildren, as well as the degree to which they do so [8,9,38].

Elementary schoolchildren are physically and cognitively immature but highly social and active, increasing their vulnerability to risk [30,37]. For instance, being less cognitively developed than adolescents and adults, elementary schoolchildren have a greater tendency to choose more dangerous routes [30]. Individual attributes such as age and gender also impact route choice. For example, as children grow older, they exhibit more independent travel behavior. Similarly, female students are often exposed to higher risk, resulting in their increased concern about safety and exhibiting less independent travel behavior than boys [1,39]. Travelling alone or with company has also been found to affect the degree of independent travel behavior.

#### 2.2.2. Factors Influencing the Selection of Non-Shortest Route

In respect to elementary school students, whose main purpose is to travel to school, route selection varies according to the travel distance to their destination [39,40,41] and degree of network connectivity at the street level [37]. According to Shatu [7], elementary school students tend to choose the shortest route as the travel distance to their destination increases, and they tend to minimize changes in direction [36,42]. Schoolchildren’s preference for a straight path has been found to depend on the friendliness of the neighborhood walking environment [6,12,32,37,43,44]. The higher the neighborhood’s walkability, the greater the likelihood that elementary schoolchildren will choose walking as a means of travel [45,46]. However, when there are excessive factors that obstruct walking, such as low street connectivity or increased traffic, children will be more likely to choose a detour [6,12,13,28].

## 3. Data and Methodology

### 3.1. Study Sites

**Figure 1:**
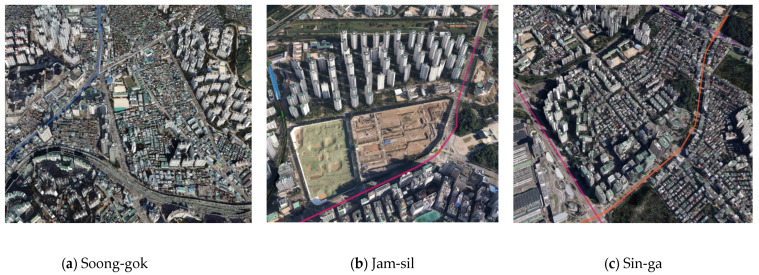
Location and surrounding status of the three study sites.

In order to analyze which environmental factors impact school route selection among elementary school students, this study examines the urban form and neighborhood environments of three elementary schools in Seoul, Korea: namely, Soong-gok Elementary School, Jam-sil Elementary School, and Sin-ga Elementary School. The scope of the research site was set based on the school district map. More specifically, Soong-gok Elementary School comprises 92.40 ha (0.92) and a surrounding area of 466 ha, Jam-sil Elementary School itself comprises an area of 35.67 ha (0.36) and a surrounding area (i.e., school district) of 1227 ha (0.80), while Sin-ga Elementary School comprises 1227 ha (0.80) and a surrounding area of 719 ha. Figure 1 presents the location and surrounding neighborhood area of the target sites are shown.

### 3.2. Spatial Structure Characteristics of Selected Study Sites

**Figure 2:**
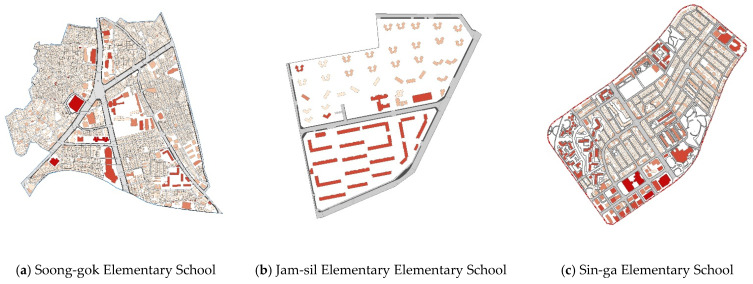
Spatial structure and shape characteristics of the study sites (GIS).

This study quantifies the pedestrian environment and network of the targets sites by examining the spatial structure in terms of the size of the block and the shape of the axis (i.e., formal/atypical) [16]. In respect to this study’s target sites, Soong-gok Elementary School possesses an irregular urban structure in which the spatial structure is naturally formed, blocks are cut off by arterial roads, and the horizontal pattern is partially mixed with a grid type and a curved irregular shape. Jam-sil Elementary School exhibits a pattern in which the spatial structure is standardized as a super grid, and the horizontal pattern is evenly distributed up, down, left, and right [21]. Finally, Sin-ga Elementary School possesses a spatial structure whereby the region is standardized as a grid, with the pattern distributed in a mixture of small-scale grid and cul-de-sac types centered on low-rise residential areas. Figure 2 presents the shape and spatial structure of the selected study sites.

In respect to the housing type of each study site, Jam-sil Elementary School possesses a high skyline in a relatively large block, with the school district comprised of apartments only (Jam-sil Elementary School is located in a large apartment complex with more than 6000 households). In contrast, the district in which Soong-gok Elementary School is situated comprises a similar ratio of apartments (42%) and detached houses (41%), with the remaining buildings consisting of apartments. Meanwhile, the district in which Sin-ga Elementary School is situated primarily comprises villas (66%), and a similar number of detached houses (16%) and apartments (17%).

Unlike the original English meaning, the term "villa" in Korea is used to describe a small apartment house with less than four floors. The term “apartment” is similar to meaning of this term in the West, and even those are relatively decent levels of apartments, and are classified into three types according to Korean law: apartments with more than five floors, apartments with less than four floors, and row houses with a total floor area of 660 m^2^. In this study, (1) a large apartment complex implies a high-rise apartment with at least 30 floors and at least 5000 households; (2) a small apartment complex refers to an apartment with less than 300 households, consisting of one or two buildings. In such apartments, villas (Korean-style villas) and several detached houses are often rebuilt. As noted, the spatial structure characteristics of neighborhood pedestrian environments differ depending on the size of the block and the shape of axis. This study examines whether there are any differences between the physical walking environment and pedestrian network surrounding elementary schools. The presence of such differences indicates that various factors play a role in the satisfaction of elementary schoolchildren and school route selection within a neighborhood environment, and that these factors and the influence thereof vary according to spatial structure.

### 3.3. Survey Data

This study conducted a survey of elementary school students attending the three selected research sites (Soong-gok, Jam-sil, and Sin-ga elementary schools). Each school was allocated 100 copies of the survey, which were randomly distributed to students in the fourth to sixth grades. The survey was conducted under the guidance of teachers at each elementary school from 15 July to 3 August 2020. A total of 273 questionnaires were retrieved; after excluding incomplete or inconsistently answered questionnaires, a total of 251 valid questionnaires were used for analysis (The survey was approved by the teacher in charge of each school student, and was conducted under the supervision of the teacher).

The questionnaire was designed to examine factors influencing respondents’ selection of school route. For this purpose, individual pedestrian characteristics and route choice (shortest route vs. non-shortest route) and neighborhood environment satisfaction (factors) were constructed as measurement items (The route selection questions were chosen by the respondents themselves). Table 1 presents the specific measurement items employed in this study.

## 4. Results

### 4.1. Survey Results

Table 2 presents the general characteristics of the respondents. With regard to gender, 50.6% of respondents were male. In terms of study site, 37.45% of respondents attended Jam-sil Elementary School students, 32.27% attended Sin-ga Elementary School, and 30.25% attended Soong-gok Elementary School. With respect to grade, 5.98% of respondents were in the 4th grade, 12.35% in the 5th grade, and 81.67% in the 6th grade. With respect to height, 39.04% of respondents were less than 4.9 ft, while 60.96% were more than 4.9 ft. With regard to housing type, 64.14% of respondents lived in apartments, while the remaining 35.86% lived in other types of housing such as single-family houses or villas.

With respect to respondent walking behavior, 39.44% of respondents reported that they walked with one or more companions, while 60.56% said they walked alone. The majority of respondents (90.84%) claimed to walk with purpose (i.e., walking as an essential activity), while just 9.16% reported walking with no purpose (i.e., optional activity). In terms of walking time, 5.98% of respondents reported a travel time of less than 5 min, 54.58% reported a travel time 5–20 min, while 37.05% reported a travel time of more than 20 min. With regard to experience of inconvenience, 21.51% of respondents reported experiencing inconvenience in their walking environment, 37.05% reported experiencing inconvenience in terms of the pedestrian network, while 41.43% purported experiencing no inconvenience.

### 4.2. Respondent Satisfaction and Route Choice

As Table 3 shows, all study sites—Jam-sil Elementary School (3.95), Sin-ga Elementary School (3.59), and Soong-gok Elementary School (3.56)—reported general satisfaction with the neighborhood walking environment. However, the overall satisfaction value was F = 5.59 (*p* < 0.01), indicating that satisfaction differed in each study site. Post-analysis revealed statistically significant differences in the satisfaction level of each measurement item for the neighborhood walking environment of each study site in all factors except “slope” and “blind spot”.

As Table 4 shows, elementary school students’ route selection depends on the region in which they live, with “shortest route” and “non-shortest-route” exhibiting differences in terms of distribution. In the case of Jam-sil Elementary School, where apartments are the dominant housing type, more respondents chose the “non-shortest route” over the “shortest route.” However, in this study, respondents’ overall distribution of path selection is not one-sided and some “shortest” routes are actually similar to the “non-shortest” depending on respondent residence. As such, this study further examines whether route selection varies depending on satisfaction with the neighborhood environment, as well as whether the factors affecting route selection vary according to the characteristics of the region.

### 4.3. Factors Influencing Route Selection

Table 5 presents the results of the logistic regression analysis of the neighborhood pedestrian environment, pedestrian network, satisfaction, individual characteristics, and spatial structure, with route selection as the dependent variable. Findings can be summarized in three points as follows. First, the analysis of the factors affecting the route choice of elementary school students revealed that the probability of choosing the shortest route increased by 52% when respondents’ satisfaction with “vehicle traffic and speed” increased by one. This means that in a pedestrian environment with many cars, there is a tendency to choose a safer detour, regardless of whether it takes longer to reach the destination. Indeed, “vehicle traffic and speed” was found to have a greater impact on route selection than other factors.

Second, the probability of choosing the shortest route was found to decrease by 32% when satisfaction with the “aesthetic impression (human scale)” increased by one. In this respect, elementary school students tended to choose a more pleasant walking environment, even if meant taking a longer route, to avoid unattractive or unpleasant environments (e.g., unpleasant odors from trash or facilities on the route).

Third, the probability of the shortest route choice was found to decrease by 39% in the presence of one or more companions. In other words, elementary school students tend to choose the shortest route to their destination when walking alone, but may choose a longer route when walking with companions if it proves more walkable for two or more pedestrians in terms of safety and comfort.

### 4.4. Analysis of Individual Areas

Table 6 presents the results of the logistic regression analysis of the neighborhood pedestrian environment, pedestrian network, satisfaction, individual characteristics, and spatial structure, with route selection as the dependent variable. Findings can be summarized in three points as follows. First, the analysis of the factors affecting the route choice of elementary school students revealed that the probability of choosing the shortest route increased by 52% when respondents’ satisfaction with “vehicle traffic and speed” increased by one. This means that in a pedestrian environment with many cars, there is a tendency to choose a safer detour, regardless of whether it takes longer to reach the destination. Indeed, “vehicle traffic and speed” was found to have a greater impact on route selection than other factors.

### 4.5. Soong-gok Elementary School

With regard to Soong-gok Elementary School, the analysis revealed that in terms of pedestrian convenience, the probability of choosing the shortest route decreased by 61% each time satisfaction with “continuity of walking space” increased by one. This can be interpreted as a tendency to choose a longer route if that route is free from facilities and illegally parked vehicles that obstruct walkability. As such, elementary school students who are more satisfied with the continuity of their walking paths are more likely to choose a detour route if it offers greater convenience.

In terms of pedestrian activity, the probability of choosing the shortest route was found to decrease by 59% every time the satisfaction level for “comport of walking space” increased by one. This indicates a tendency to take a minor detour to maintain a comfortable space environment and avoid issues such as trash or odor.

In terms of obstacles to walking, the probability of choosing the shortest route was found to increase by 1.3 times whenever satisfaction with “illegal parking” increased by 1, and 1.8 times whenever satisfaction with “vehicle traffic and speed” increased by 1. This finding is related to the spatial structure of the school’s area, in which blocks are cut off due to main roads and horizontal patterns are mixed with grid-shaped and curved irregularities. This spatial structure means that routes to Soong-gok Elementary School may be characterized by a greater difference between direct distance and actual walking distances compared to other elementary schools. This indicates that if there are fewer external risk factors, such as vehicles, and if satisfaction increases, then students will tend to choose the shortest path because it is more direct and economical.

### 4.6. Jam-sil Elementary School

With regard to Jam-sil Elementary School, analysis revealed that in terms of pedestrian activity, the probability of choosing the shortest route decreased by 60% as satisfaction when “aesthetic impression (human scale)” increased by one. This can be interpreted as a tendency to take a minor detour to a destination if it is more aesthetically pleasing and pleasant.

In terms of obstacles to walking, the probability of choosing the shortest route was found to increase 1 time whenever satisfaction with “road slope” increased by 1, and 2.3 times when “vehicle traffic and speed” increased by 1. This finding is related to the spatial structure of the Jam-sil Elementary School area insofar as it comprises a super grid formed around main roads, and a pedestrian space that is evenly distributed from side to side on flat land without slope. With no or gentle slope and fewer risk factors from vehicles, children are less likely to take detours and more likely to opt for the fastest route.

With respect to individual characteristics, the probability of choosing the shortest route was found to decrease by 70% if the respondent had company when traveling to school. This indicates a tendency to choose a more comfortable but longer route when traveling with a companion and the shortest route when traveling alone.

### 4.7. Sin-ga Elementary School

Results of regression analysis of Sin-ga Elementary School can be summarized in seven points as follows. First, in terms of pedestrian safety, the probability of choosing the shortest route was found to decrease by 58% as satisfaction with “safety (collision avoidance)” increased by one. In other words, students tended to choose a detour if it was considered to be safer in terms of the potential risk of collision from external hazards (e.g., vehicles, motorcycles, and bicycles).

Second, in terms of pedestrian convenience, the probability of choosing the shortest route was found to increase by 3.4 times whenever satisfaction with “continuity of walking space” increased by 1, and 3.1 times when “legibility of wayfinding” increased by 1. As such, a lack of obstacles to walking (e.g., illegally parked vehicles) and obstructions (e.g., blind spots) negated the need for a detour—that is, students opted for the shortest and fastest route providing it was considered convenient and free of obstruction.

Third, in terms of obstacles to walking, the probability of choosing the shortest route was found to decrease by 57% whenever satisfaction with “illegal parking” increased by one. This indicates a tendency to choose a longer and/or less direct route if it is considered to have fewer external threats in the form of illegally parked vehicles.

Fourth, in terms of accessibility, the probability of choosing the shortest route was found to decrease by 67% whenever satisfaction with the “accessibility of cultural and welfare facilities (e.g., children’s libraries)” increased by one. This indicates a tendency to choose a route with cultural and/or welfare facilities, particularly when travelling from school to home.

Fifth, the probability of choosing the shortest route was found to increase by 10.6 times whenever “satisfaction of walking environment” increased by one. This indicates that the higher the overall satisfaction with neighborhood walking environment, the greater the likelihood of choosing the shortest and fastest route.

Sixth, in terms of housing type, analysis revealed that the probability of choosing the shortest route decreased by 90% if the housing type is not “apartment.” This finding is closely related to the standardized grid spatial structure. Moreover, the small-scale grid-shaped horizontal patterns in low-rise residential areas—such as that of Sin-ga Elementary School—are closely connected to one another, resulting in high transmittance. In this case, there is little difference between the direct distance and actual walking distance, producing a greater variety of potential routes. In such instances, route selection varies due to environmental factors.

Finally, in terms of individual characteristics, the probability of choosing the shortest route was found to decrease by 90% when traveling to school with one or more companions. This indicates a tendency to choose a comfortable route when traveling with a companion, rather than traveling alone. As a result of the spatial structure of the Sin-ga Elementary School area, which comprises small blocks, there are many walkable and convenient routes from which to choose, producing a greater tendency to choose the “non-shortest path” when walking with companions. In contrast, the spatial structure of areas such as that of Soong-gok Elementary School—in which the natural environment impedes road and block connectivity—limits the number of potential routes and does not facilitate multipurpose walking. In such environments, company does not affect route choice.

## 5. Discussion

This study was conducted on elementary school students in three neighborhood living areas with different urban structures, densities, and street patterns. In the survey, with the cooperation of each elementary school, 251 valid samples were obtained by examining students’ perceptions and preferences of path selection and walking environment. For accuracy and convenience purposes, the study was conducted on elementary school students in senior grades (grades 4, 5, and 6) with sufficient explanation from the teacher.

There are four main points to discuss following the results obtained. First, among the distribution ratios of path selection in elementary school students, the proportion of choosing the non-shortest path was high at 41.83% (n = 105). This finding contradicts the general tendency of pedestrians to prefer the shortest route [15,34]. Most pedestrians consciously choose paths due to the various factors that affect them [12], and generally prefer the shortest route. Based on Gehl’s theory that pedestrians stick to this preference except when faced with danger, it can be implied that many elementary school students in the neighborhood are exposed to danger.

Second, the ratio of non-shortest path selection gradually decreased in the order of Jam-sil (47.87%), Soong-gok (44.74%), and Sin-ga (32.10%) elementary schools. These sites also showed different influences on path selection, and the ratio of shortest path selection differed. This finding supports Jacobs’ theory [47] (the advantage of small blocks) that the horizontal pattern is formalized and the smaller the block size, the higher the shortest path selection ratio. Increasing the satisfaction with "continuity of walking space" by one level reduces the probability that Soong-gok (1) will choose the shortest path, and Sin Price (3) will choose the shortest path. This is because horizontal patterns made of small grid-shaped blocks are highly permeable due to dense walking paths. If the permeability is high, there is a small gap between the straight distance and the walking distance, and various paths can be selected [36,47].

Third, some studies on the purpose of walking have shown that 80% of pedestrians choose the shortest path, and this choice decreases when they engage in no-purpose activities [14,15]. This implies that in the case of purpose activities, the ratio of selecting shortest paths increases [11,12,14]. In this study, even though 90.84% of respondents chose purpose walking, non-shortest path selection was higher than 40% (Table 2). This is contrary to what previous research [14,15] has claimed, and can be seen as a reflection of the tendency of pedestrians to select a safer path away from surrounding risk factors. For example, pedestrians tend to prefer sidewalks to avoid the risk of a car crash rather than mixed traffic streets (without sidewalks). In the case of residential areas in Seoul, the majority of roads are mixed traffic streets (the ratio of mixed traffic streets that are less than 12 m wide is 77.4%, which can be interpreted as a sharp difference in safety from commercial areas). Efforts to secure safe walking spaces in residential areas have recently been pursued through projects such as pedestrian priority roads; however, there is still a paucity of such projects. In particular, illegal parking and vehicle speed have a significant impact in area (small blocks) with mostly mixed traffic streets. This is worthy of attention because it is the biggest factor that causes elementary school students to bypass their destinations.

Fourth, company is an important factor that influences elementary school students’ path selection. The Sin-ga neighborhood has several small blocks that provide many opportunities to choose various routes, and it is easy to engage in a multipurpose walk and visit multiple destinations, which increases the ratio of selection of non-shortest routes depending on the presence or absence of a companion. However, the Soong-gok neighborhood is cut off by the boulevard and due to naturally occurring street patterns, it is difficult to engage in multipurpose walks due to high detours. Furthermore, it can be interpreted as selecting a path regardless of the presence of a companion because the difference between the straight distance and the actual walking distance is significant. In neighborhoods where multipurpose walking is likely, it is necessary to provide a route for elementary school students to walk safely through the creation of “pedestrian priority roads projects” or “school zones” to assess public buildings (public libraries, parks, etc.).

## 6. Conclusions

The findings of this study have two main implications, specifically in respect to bypass index. As noted, depending on the spatial structure characteristics of the neighborhood environment, factors influencing elementary school students’ travel route selection may be diverse or limited. First, when the difference between the direct and actual walking distance increases (i.e., when the bypass index increases), the economic and utility of walking decreases. This has a significant implication as it can be used to determine the main factors affecting route selection among the target group based on the characteristics of spatial structure, as well as how the behavioral characteristics and preferences of these students influence these factors. Accordingly, future research is necessary to further define and evaluate these characteristics and their impact on route selection in an expanded range of target types.

Second, in the case of little deviation between the direct distance and actual walking distance (i.e., when the transmittance index is high), the economy and efficiency of walking are enhanced. However, the results indicate that this factor is further shaped by various factors in the neighborhood environment. Route choice can also be influenced by the desire to visit multiple destinations or when walking with friends. This implies that route selection will vary in accordance with the extent to which the economic and practical utility of walking is guaranteed. Future research is needed to clarify these characteristics, particularly as their influence and relationships with other factors may vary.

The findings of this study can also be used to facilitate the development of living areas in which elementary school students can avoid vehicle-oriented environments, thereby encouraging active walking activities for the socially vulnerable groups by life cycle.

## Figures and Tables

**Table 1:** Survey measurement factors.

Division	Measures	Variable Description
Personal characteristics	Gender, age (grade), companionship, housing type, purpose of walking, walking distance, route choice (shortest vs. non-shortest)	Survey (five-point Likert scale)
Satisfaction with neighborhood environment (factors)	Pedestrian safety, pedestrian convenience, pedestrian activity, obstacles to walking, accessibility, CPTED (safety facilities, dead zone, evacuation facilities), satisfaction (walking environment and pedestrian network)	Survey (five-point Likert scale)

**Table 2:** Demographic characteristics of the respondents of the study (n = 251).

Classification		Frequency (Persons)	Ratio (%)	Classification		Frequency (Persons)	Ratio (%)
Gender	Male (0)	127	50.60	Grade	4th grade (0) 5th grade (1)	15	5.98
	Female (1)	124	49.40		6th grade (3)	31 205	12.35 81.67
Region (School name)	Soong-gok Elementary (1)	76	30.28	Height	Under 4.9 ft (0)	98	39.04
	Jam-sil Elementary School (2)	94	37.45		More than 4.9 ft (1)	153	60.96
	Sin-ga Elementary School (3)	81	32.27	Walking time	Within 5 min (0)	15	5.98
Company	None (0)	152	60.56		More than 5 min and Within 20 min (1)	137	54.58

**Table 3:** Differences in satisfaction by study site (n = 251).

Characteristics	Soong-gok Elementary School (*n* = 76)		Jam-sil Elementary School (*n* = 94)		Sin-ga Elementary School (*n* = 81)		F	Post-Hoc Analysis (Scheffe Test)
	Mean	SD	Mean	SD	Mean	SD		
Overall satisfaction	3.56	0.8	3.95	0.8	3.59	0.9	5.59 ** ^1^	2, 3 > 1
Pedestrian safety	3.10	1.2	3.58	1.2	3.24	1.3	3.53 **	2, 3 > 1
Pedestrian convenience	2.75	1.2	3.37	1.2	3.20	1.3	5.36 **	2, 3 > 1
Pedestrian activity	3.30	0.9	3.82	1.1	3.29	1.3	6.10 **	1, 2 > 3
Road slope	3.43	1.0	3.82	1.1	3.51	1.2	2.91	2, 3 > 1
Illegal parking	3.13	1.1	3.61	1.1	3.18	1.3	4.57 *	2, 3 > 1
Accessibility of spatial facilities	4.01	1.1	4.47	0.8	4.4	0.8	6.41 **	2, 3 > 1
Accessibility of school	3.92	1.3	4.46	0.8	4.30	0.9	6.23 **	2, 3 > 1
Dead (remote) zone(s)	3.46	1.3	3.85	1.2	3.53	1.3	2.48	2, 3 > 1
Evacuation facilities	3.28	1.2	3.91	1.2	3.35	1.4	6.64 **	2, 3 > 1

^1^ * *p* < 0.05, ** *p* < 0.01.

**Table 4:** Route selection among elementary school students by residential area (n = 251).

Case	Route Selection Characteristics					x^2^
	Overall (%)	Non-Shortest Route Selection		Shortest Route Selection		
		Frequency	Ratio (%)	Frequency	Ratio (%)	
Soong-gok Elementary School (1)	(100%)	34	44.74	42	55.26	4.8266
Jam-sil Elementary School (2)	(100%)	45	47.87	49	52.13	
Sin-ga Elementary School (3)	(100%)	26	32.10	55	67.90	
Total	251 (100%)	105	-	146	-	

**Table 5:** Logistic regression results (n = 251) ^1^.

Categories		Variables	z	S. E	P	Odds Ratio
		Constant				1.992
Neighborhood pedestrian environment (walkability)	Pedestrian safety	Sufficiency of walking space	0.28	0.172	0.776	1.048
		Availability of walking space	−0.84	0.156	0.402	0.858
		Safety (collision avoidance)	−0.31	0.162	0.757	0.949
	Pedestrian convenience	Connectivity of walking space	0.77	0.192	0.442	1.139
		Continuity of walking space	0.63	0.188	0.528	1.112
		Legibility of wayfinding	1.04	0.226	0.298	1.214
	Pedestrian activity	Comfort of walking space	−1.09	0.148	0.276	0.822
		Aesthetic impression (human scale)	−2.6	0.100	0.009 ***	0.684
		Diversity and interests (e.g., pocket parks, open spaces, and public spaces)	−1.03	0.126	0.301	0.859
Pedestrian network	Obstacles to walking	Road slope	−0.37	0.146	0.712	0.945
		Illegal parking	0.69	0.204	0.487	1.133
		Vehicle traffic and speed	2.46	0.261	0.014 **	1.525
	Accessibility	Accessibility of spatial facilities (e.g., parks, public spaces, plazas)	−0.54	0.192	0.592	0.891
		Accessibility of schools (including kindergartens)	−1.01	0.154	0.314	0.830
		Accessibility of cultural and welfare facilities (e.g., children’s library)	−0.43	0.140	0.669	0.938
	CPTED	Safety facilities (e.g., CCTV, streetlights, emergency bells)	0.4	0.168	0.69	1.065
		Dead zone(s) (i.e., blind spots)	−0.79	0.149	0.429	0.873
		Evacuation facilities (e.g., police substations, escape routes)	0.76	0.202	0.449	1.143
Satisfaction		Satisfaction of route choice	0.97	0.328	0.334	1.280
Individual	Gender	Gender (Male = 0, Female = 1)	−1.45	0.193	0.148	0.651
	Housing type	Apartment (No = 0, Yes = 1)	−0.32	0.314	0.752	0.895
	Company	Company (No = 0, Yes = 1)	−1.67	0.181	0.096 *	0.609
Spatial structure	Region	Region 2 * (Jam-sil Elementary School)	−0.31	0.370	0.758	0.878
		Region 3 * (Sin-ga Elementary School)	1.68	0.735	0.092 *	1.911

^1^ Dependent variable: route selection (shortest route = 1), *** *p* < 0.01, ** *p* < 0.05, * *p* < 0.1; R^2^ = 0.262; x^2^ = 34.10 (0.025 **).

**Table 6:** Logistic regression results ^1^.

Categories	Variables	Soong-gok Elementary School (n = 76)			Jam-sil Elementary School (n = 94)			Sin-ga Elementary School (n = 82)			
		z	p	Odds	z	p	odds	z	p	odds	
Pedestrian safety	Sufficiency of walking space	−1.43	0.152	0.543	1.63	0.103	1.781	−1.4	0.160	0.512	
	Availability of walking space	0.67	0.503	1.400	−1.62	0.106	0.530	0.33	0.743	1.170	
	Safety (collision avoidance)	0.19	0.853	1.074	0.63	0.530	1.248	−1.71	0.087 *	0.420	
Pedestrian convenience	Connectivity of walking space	−0.35	0.729	0.855	−0.39	0.694	0.878	0.18	0.861	1.080	
	Continuity of walking space	−1.94	0.052 *	0.386	0.08	0.933	1.029	2.33	0.02 **	4.495	
	Legibility of wayfinding	−1.18	0.239	0.578	−1.22	0.224	0.613	2.44	0.015 **	4.186	
Pedestrian activity	Comfort of walking space	−1.93	0.053 *	0.415	−0.85	0.396	0.723	−0.95	0.344	0.570	
	Aesthetic impression (human scale)	0.15	0.880	1.054	−2.46	0.014 **	0.398	−1.24	0.214	0.658	
	Diversity and interests (e.g., pocket parks, open spaces, public spaces)	−0.61	0.543	0.787	−0.54	0.590	0.843	−0.71	0.476	0.785	
Obstacles to walking	Road slope	−1.46	0.144	0.562	1.89	0.059 *	2.021	−0.29	0.769	0.889	
	Illegal parking	1.66	0.097 *	2.352	1.01	0.313	1.474	−1.71	0.087 *	0.434	
	Vehicle traffic and speed	2.49	0.013 **	2.842	3.02	0.003 ***	3.323	−0.81	0.416	0.685	
Accessibility	Accessibility of spatial facilities (e.g., parks, public spaces, plazas)	−1.33	0.182	0.555	0.4	0.693	1.204	−0.8	0.424	0.555	
	Accessibility of schools (including kindergartens)	0.51	0.610	1.204	−0.41	0.679	0.832	−1.09	0.276	0.485	
	Accessibility of cultural & welfare facilities (e.g., children’s library)	0.26	0.792	1.109	0.08	0.934	1.026	−1.77	0.076 *	0.331	
CPTED	Safety facilities (e.g., CCTV, streetlights, emergency bells)	0.36	0.720	1.172	−0.21	0.833	0.926	1.57	0.117	2.076	
	Dead zone(s) (i.e., blind spots)	−0.65	0.513	0.765	−0.46	0.648	0.851	−0.38	0.705	0.849	
	Evacuation facilities (e.g., police substations, escape routes)	−0.49	0.622	0.800	1.45	0.147	1.702	0.16	0.876	1.073	
Satisfaction	Satisfaction of route selection	1.34	0.181	2.663	−1.13	0.260	0.519	2.33	0.020 **	11.599	
Gender	Gender (Male = 0, Female = 1)	−1.1	0.273	0.440	−1.26	0.206	0.469	0.57	0.571	1.591	
Housing type	Apartment (No = 0, Yes = 1)	−0.25	0.802	0.839	0.55	0.585	1.907	−2.26	0.024 **	0.102	
Company	Company (No = 0, Yes= 1)	0.91	0.361	1.905	−2	0.046 **	0.298	−1.99	0.046 **	0.187	

^1^ Dependent variable: route selection (shortest route = 1). *** *p* < 0.01, ** *p* < 0.05, * *p* < 0.1; R^2^ = 0.262; x^2^= 34.10(0.025 **). (1) Soong-gok Elementary School: R^2^ = 0.284, x^2^ = 29.71(0.125), (2) Jam-sil Elementary School: R^2^ = 0.269, x^2^ = 35.07(0.0381 **), (3) Sin-ga Elementary School: R^2^ = 0.360, x^2^ = 36.61(0.0261).

## Data Availability

Not applicable.
